# Diagnostic Accuracy of Serum‐Based Fibrosis Scores for Clinically Significant Liver Fibrosis in an East Asian Cohort

**DOI:** 10.1002/kjm2.70296

**Published:** 2026-09-24

**Authors:** Sheng‐Feng Lin, Chun‐Chao Chang

**Affiliations:** ^1^ Department of Public Health, School of Medicine, College of Medicine Taipei Medical University Taipei Taiwan; ^2^ School of Public Health, College of Public Health Taipei Medical University Taipei Taiwan; ^3^ Department of Emergency Medicine Taipei Medical University Hospital Taipei Taiwan; ^4^ Center of Evidence‐Based Medicine Taipei Medical University Hospital Taipei Taiwan; ^5^ Department of Medical Research Taipei Medical University Hospital Taipei Taiwan; ^6^ Division of Gastroenterology and Hepatology, Department of Internal Medicine Taipei Medical University Hospital Taipei Taiwan; ^7^ Division of Gastroenterology and Hepatology, Department of Internal Medicine, School of Medicine, College of Medicine Taipei Medical University Taipei Taiwan; ^8^ TMU Research Center for Digestive Medicine Taipei Medical University Taipei Taiwan

**Keywords:** diagnostic accuracy, FibroScan, liver fibrosis, noninvasive fibrosis assessment, serum fibrosis scores

## Abstract

Serum‐based fibrosis scores are widely used for noninvasive risk stratification in chronic liver disease; however, direct comparisons across liver disease etiologies remain limited, particularly in Asian populations with a high prevalence of hepatitis B virus (HBV) and hepatitis C virus (HCV) infection. This study retrospectively analyzed the data of adults who underwent transient elastography (FibroScan) at a university‐affiliated hospital. Three fibrosis scores were evaluated: Fibrosis‐4 index (FIB‐4), aspartate aminotransferase‐to‐platelet ratio index (APRI), and metabolic dysfunction‐associated fibrosis‐5 (MAF‐5). Associations with advanced fibrosis (F3–F4) were examined using logistic regression. Discrimination was assessed using the area under the receiver operating characteristic curve (AUROC). Of the 2355 participants, the etiologic subgroups included metabolic dysfunction–associated steatotic liver disease (MASLD; *n* = 1070), HBV infection (*n* = 1459), and HCV infection (*n* = 250); 10.4% of the participants had advanced fibrosis (F3–F4). In the overall cohort, all scores were significantly associated with advanced fibrosis (*p* < 0.001) and demonstrated similar overall discrimination with AUROC in a range of 0.824–0.839. MAF‐5 had the highest AUROC (0.839; 95% confidence interval [CI], 0.811–0.867) and greater risk classification compared with FIB‐4 (net reclassification improvement = 0.168; 95% CI, 0.093–0.244). Subgroup analysis revealed that MAF‐5 exhibited consistent discrimination across etiologies, with AUROCs of 0.822 (0.781–0.863) in MASLD, 0.843 (95% CI, 0.804–0.882) in HBV, and 0.810 (95% CI, 0.735–0.886) in HCV. Commonly used serum‐based fibrosis scores exhibited comparable discrimination for advanced fibrosis. However, the metabolically informed MAF‐5 score modestly improved risk classification across diverse liver disease etiologies.

## 1 Introduction

Fibrosis stage is the principal determinant of long‐term prognosis in chronic liver diseases, with mortality and hepatic complications increasing as fibrosis progresses [1]. In patients with metabolic dysfunction–associated steatotic liver disease (MASLD), the all‐cause mortality increases from 0.32 deaths per 100 person‐years in those with stages F0–F2 fibrosis to 1.76 in those with stage F4 fibrosis, with F4 fibrosis conferring more than a threefold higher risk of all‐cause mortality compared with F0 disease [1, 2, 3]. A similar prognostic gradient has been reported in patients with chronic hepatitis B virus (HBV) and hepatitis C virus (HCV) infection [4] in whom fibrosis severity independently predicts hepatic events and mortality.

Although liver biopsy has traditionally been used as the histologic standard for fibrosis staging, its invasiveness, cost, sampling variability, and limited repeatability restrict its use for longitudinal monitoring and population‐level assessment [5] Accordingly, contemporary care pathways have increasingly relied on sequential noninvasive assessments, with serum‐based fibrosis scores serving as first‐line triage tools followed by elastography for secondary risk assessment. Vibration‐controlled transient elastography (VCTE; FibroScan, Echosens, Paris, France) provides a rapid, noninvasive measurement of liver stiffness as a surrogate of fibrosis severity and has been widely incorporated into clinical practice [6]. VCTE has demonstrated high performance in detecting advanced fibrosis in different liver disease etiologies, and contemporary guidelines recommend elastography as a central component of noninvasive fibrosis assessment after initial serum‐based risk stratification [7, 8, 9].

Several serum‐based fibrosis indices have been developed for clinical use (Table 1). For example, Fibrosis‐4 (FIB‐4) has been widely adopted as a first‐line triage tool because of its simplicity and reproducibility [10]. The aspartate aminotransferase‐to‐platelet ratio index (APRI) offers a readily calculable alternative based on routine laboratory parameters, although its performance may vary across different disease etiologies [11]. More recently, metabolic dysfunction–associated fibrosis‐5 (MAF‐5) was developed to incorporate metabolic and inflammatory components that better reflect contemporary disease phenotypes [12]. However, few studies have directly compared established and emerging serum‐based fibrosis scores within the same cohort by using a uniform elastography‐based reference standard.

**TABLE 1:** Formulas and components of serum‐based fibrosis scoring systems.

Scores	Variables required	Formula	Notes/units	Study population and origin
FIB‐4	Age, AST, ALT, platelet count	(Age × AST)/(platelet count × √ALT)	AST/ALT in U/L; platelets in ×10^9^/L	HIV/HCV coinfection cohort, the USA.
APRI	AST, platelet count	[(AST/AST ULN) × 100]/platelet count	AST ULN = 40 U/L; platelet count in ×10^9^/L	HCV cohort, the USA.
MAF‐5	Waist circumference, BMI, DM, AST, Platelets	−11.3674 + 0.0282 × waist circumference − 0.1761 × BMI + 0.0019 × (waist circumference × BMI) + 2.0762 × DM + 2.9207 × ln(AST) − 0.0059 × platelet count	Platelet count in ×10^9^/L	Population with metabolic dysfunction; the Netherlands

*Note:* AST and ALT levels were reported in U/L. The natural logarithm is denoted as ln().

Abbreviations: ALT, alanine aminotransferase; APRI, Aspartate Aminotransferase‐to‐Platelet Ratio Index; AST, aspartate aminotransferase; BMI, body mass index; DM, diabetes mellitus (or impaired fasting glucose where applicable); FIB‐4, Fibrosis‐4 index; MAF‐5, metabolic dysfunction–associated fibrosis‐5 score; ULN, upper limit of normal.

This knowledge gap is particularly relevant in Asia, where chronic viral hepatitis remains a major contributor to liver disease and the prevalence of MASLD is still increasing. The etiologically diverse patient population in Asia may differ substantially from the Western population for which these fibrosis scores were originally developed. To address this gap, we conducted a real‐world retrospective study in which we compared the diagnostic accuracy of three commonly used serum‐based fibrosis scores, namely FIB‐4, APRI, and MAF‐5, by using FibroScan‐defined fibrosis as the reference standard. By evaluating the performance of these scores in a cohort with diverse liver disease etiologies, we clarified their clinical utility and generalizability within contemporary care pathways that incorporate elastography.

## 2 Methods

### 2.1 Study Population and Design

This retrospective observational study was conducted at Taipei Medical University Hospital, a university‐affiliated tertiary medical center located in Taiwan. Adult patients who underwent VCTE as part of routine clinical care between January 1, 2024, and December 31, 2024, were considered to be eligible for inclusion. Clinical characteristics, laboratory results, and imaging data were obtained from electronic medical records. Laboratory measurements used to calculate fibrosis scores were obtained within 30 days of VCTE examination as part of the same clinical evaluation.

Patients with complete VCTE examination data and sufficient clinical and laboratory information to calculate at least one serum‐based fibrosis score were included. However, patients with incomplete VCTE data or missing fibrosis outcome data were excluded. Missing laboratory variables required for fibrosis score calculation were addressed using multiple imputation, as described in Section 2.5. The study protocol was approved by the Joint Institutional Review Board of Taipei Medical University (approval number: N202509023). Because of the retrospective nature of the study, the requirement for written informed consent was waived by the institutional review board. This study was conducted in accordance with the Declarations of Helsinki.

### 2.2 Serum‐Based Scoring Systems

We compared the following serum‐based fibrosis scores: FIB‐4, APRI, and MAF‐5. FIB‐4 has been widely validated as a first‐line noninvasive marker of liver fibrosis in patients with chronic liver disease [10]. APRI was developed as a simple laboratory‐based index for fibrosis assessment [13]. MAF‐5 is a recently developed noninvasive fibrosis score that has been externally validated in patients with metabolic dysfunction–associated liver disease [12]. In addition to analyzing MAF‐5 as a continuous score, we applied the following published MAF‐5 fibrosis risk categories: low risk, < 0; intermediate risk, 0–1; and high risk, ≥ 1 [12]. All fibrosis scores were recalculated within each imputed dataset using standardized formulas to ensure consistency across analyses (Table 1).

### 2.3 Assessment of Liver Fibrosis by Using VCTE

Before VCTE, trained staff measured waist circumference with the participants standing upright, with their feet together, and arms relaxed at their sides. A nonstretchable tape was positioned horizontally midway between the lower margin of the last palpable rib and the superior border of the iliac crest, and the measurements were obtained at the end of normal expiration without compressing the skin [14].

Liver fibrosis severity was assessed using VCTE (FibroScan, Echosens, Paris, France), performed during routine clinical care by trained operators in accordance with the manufacturer's standard recommendations. Liver stiffness measurements are expressed in kilopascals (kPa). Examinations were considered to be reliable if at least 10 valid measurements were obtained with an interquartile range (IQRs) to median ratio of ≤ 30%. Fibrosis stages were defined using established liver stiffness measurement cutoffs as follows: F0 (no fibrosis), F1–F2 (mild to moderate fibrosis), F3 (advanced fibrosis), and F4 (cirrhosis). In the primary analyses, F3–F4 was classified as advanced fibrosis, whereas F0–F2 was classified as nonadvanced fibrosis, consistent with previous validation studies [15, 16, 17].

### 2.4 Etiology Classification

HBV infection was defined by serum hepatitis B surface antigen positivity, whereas current HCV infection was defined by detectable serum HCV RNA. Alcohol‐associated liver disease was identified on the basis of the clinical diagnosis recorded in the patient's electronic medical record. Steatotic liver disease was operationally defined as hepatic steatosis determined by the controlled attenuation parameter at a prespecified threshold of ≥ 248 dB/m measured during VCTE, corresponding to the optimal cutoff for detecting hepatic steatosis of grade S1 or higher [18]. MASLD was defined as steatotic liver disease plus at least one cardiometabolic risk factor, consistent with current multisociety consensus criteria [19, 20, 21].

Cardiometabolic risk factors included overweight or obesity (body mass index ≥ 23 kg/m^2^), increased waist circumference (≥ 90 cm in men and ≥ 80 cm in women), diabetes mellitus, hypertension, hypertriglyceridemia (triglycerides ≥ 150 mg/dL), or reduced high density lipoprotein cholesterol (< 40 mg/dL in men and < 50 mg/dL in women) [20, 21]. Because MASLD may coexist with other chronic liver diseases, MASLD was not considered mutually exclusive with HBV, HCV, or alcohol‐associated liver disease. For patients with overlapping etiologies, VCTE‐defined advanced fibrosis was classified using the liver stiffness threshold corresponding to the documented primary liver disease; HBV‐specific thresholds were additionally stratified by ALT level. Etiology‐specific VCTE‐derived liver stiffness thresholds, based on the American Association for the Study of Liver Disease practice guidelines and representative studies, are provided in Table S1 [9, 16, 18, 19, 20, 21, 22].

### 2.5 Statistical Analysis

The normality of continuous variables was assessed using the Shapiro–Wilk test. Continuous variables were summarized as medians with IQRs, and were compared between groups using the Mann–Whitney *U* test. Categorical variables were summarized as counts and percentages and were compared using the Pearson's *χ* ^2^ test or Fisher's exact test, as appropriate. To account for missing covariate data, we performed multiple imputation with chained equations under the missing‐at‐random assumption (Table S2). Simple logistic regression models were used to estimate the diagnostic performance of each fibrosis score for detecting advanced liver fibrosis or cirrhosis (F3–F4). Diagnostic performance is expressed as the area under the receiver operating characteristic curve (AUROC) with 95% confidence intervals (CIs). The dependent variable was the presence of advanced liver fibrosis or cirrhosis (F3–F4), and the independent variable was each fibrosis score. For MAF‐5, we additionally assessed the prevalence of advanced fibrosis across the original MAF‐5 categories (< 0, 0–1, and ≥ 1) [12]. Category‐specific sensitivity, specificity, positive predictive value, negative predictive value, and accuracy were calculated for each risk category.

Category‐free net reclassification improvement (cfNRI) was calculated to compare the predictive performance of the models by determining the net proportion of individuals whose predicted risk shifted in the correct direction when alternative models were compared with the reference model, without using predefined risk categories [23]. FIB‐4 was selected as the reference for evaluating the incremental predictive performance of alternative fibrosis scores because of its simplicity. All discrimination and reclassification analyses were conducted in the overall cohort and in prespecified subgroups in accordance with sex, MASLD, HBV infection, and HCV infection. A two‐sided *p* value of < 0.05 was considered statistically significant. All statistical analyses were conducted using the SAS software (version 9.4; SAS Institute, Cary, NC, USA).

### 2.6 Sensitivity Analysis

To evaluate the influence of missing data, sensitivity analysis I was restricted to patients with nonmissing data on VCTE‐defined advanced fibrosis status, platelet count, and all three fibrosis scores (FIB‐4, APRI, and MAF‐5). Model performance and head‐to‐head cfNRI comparisons were repeated in the overall cohort and in prespecified sex‐ and etiology‐specific subgroups. Estimates were calculated directly from the observed complete cases, and global model fit was evaluated using likelihood‐ratio *χ* ^2^ tests. Sensitivity analysis II was conducted to evaluate patients with MASLD without coexisting HBV or HCV infection. Estimates were separately calculated within each of the 50 imputed datasets and pooled using Rubin's rules; global model fit was assessed using pooled Wald *χ* ^2^ tests.

## 3 Results

### 3.1 Patient Characteristics

**FIGURE 1:**
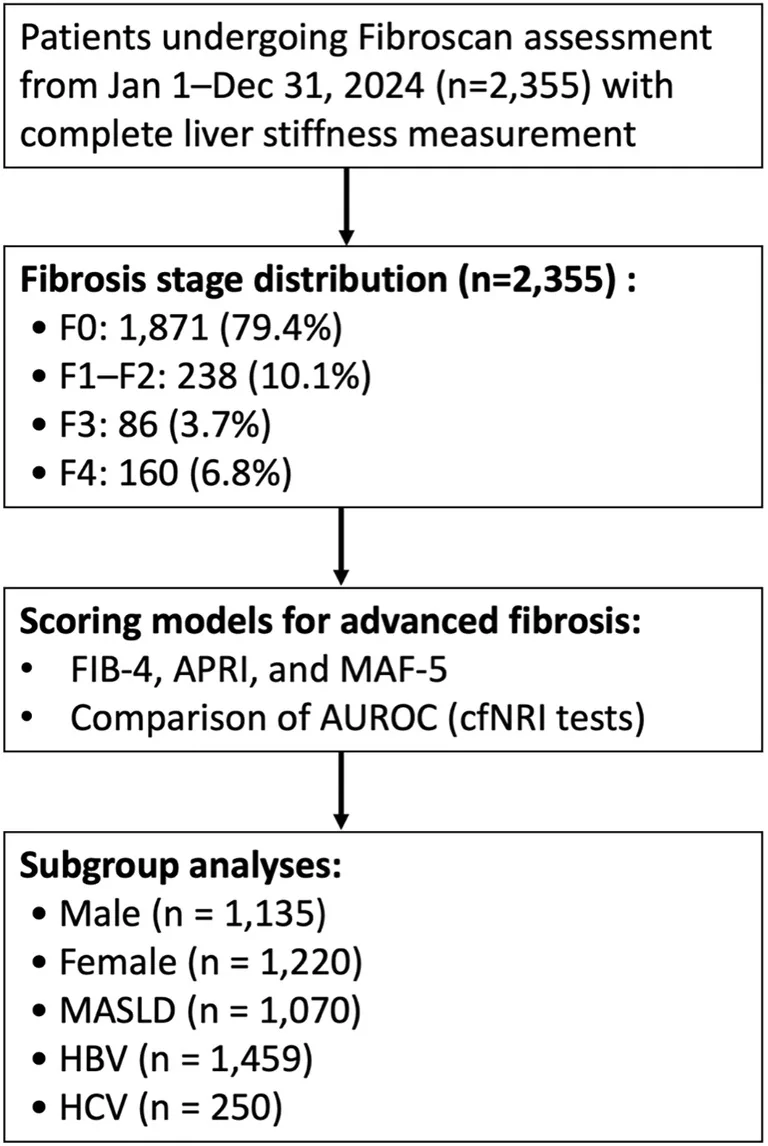
Study flow diagram.

Figure 1 presents a flow diagram of the patient selection process. Among the 2355 included patients, fibrosis stages were distributed as follows: F0, 1871 patients; F1–F2, 238 patients; F3, 86 patients; and F4, 160 patients. Table 2 presents the baseline sample characteristics stratified by liver fibrosis severity. Overall, 2109 patients (89.6%) had nonadvanced fibrosis (F0–F2), whereas 246 patients (10.4%) had advanced fibrosis (F3–F4). Patients with advanced fibrosis were significantly older than those without advanced fibrosis (median age: 64.0 vs. 55.0 years, *p* < 0.001), with a smaller proportion being women (40.7% vs. 53.1%, *p* < 0.001). Measures of adiposity, including body mass index and waist circumference, were also significantly higher in the advanced fibrosis group (all *p* < 0.001). None of the included patients underwent liver biopsy.

**TABLE 2:** Characteristics of the participants categorized by liver fibrosis severity (*N* = 2355).

Variables	Nonadvanced liver fibrosis (F0–F2)	Advanced liver fibrosis (F3–F4)	*p*
Number	2109	246	
Demographic characteristics			
Female sex, %	53.1% (1120/2109)	40.7% (100/246)	< 0.001*
Age, years	55.0 (47.0–64.0)	64.0 (54.0–71.0)	< 0.001*
BMI, kg/m^2^	24.2 (21.9–26.8)	25.7 (23.2–30.4)	< 0.001*
Waist circumference, cm	83.0 (76.5–90.0)	91.0 (83.0–101.0)	< 0.001*
Cardiometabolic risk factors^a^			
Overweight or obesity, %	62.9% (1325/2108)	76.5% (186/243)	< 0.001*
Increased waist circumference, %	45.2% (951/2103)	66.7% (156/234)	< 0.001*
Diabetes mellitus, %	10.6% (223/2109)	36.2% (89/246)	< 0.001*
Hypertension, %	23.7% (500/2109)	42.7% (105/246)	< 0.001*
Hypertriglyceridemia, %	18.1% (298/1645)	20.9% (40/191)	0.340
Reduced HDL cholesterol, %	17.1% (233/1361)	36.6% (60/164)	< 0.001*
Dyslipidemia, %	30.3% (426/1405)	48.0% (83/173)	< 0.001*
At least one cardiometabolic risk factor	75.2% (1587/2109)	90.2% (222/246)	< 0.001*
Etiology and steatosis			
MASLD, %	44.5% (939/2109)	53.3% (131/246)	0.009*
Hepatitis B viral infection, %	63.3% (1336/2109)	50.0% (123/246)	< 0.001*
Hepatitis C viral infection, %	10.0% (211/2109)	15.9% (39/246)	0.005*
HIV infection, %	0.1% (2/2109)	0.4% (1/246)	0.282
Alcohol‐associated liver disease	0.2% (4/2109)	4.9% (12/246)	< 0.001*
Controlled attenuation parameter, dB/m	242.0 (210.0–287.0)	255.5 (210.0–307.0)	0.022*
Liver stiffness measurement, kPa	4.7 (4.0–5.6)	15.1 (11.6–23.3)	< 0.001*
Laboratory tests			
Aspartate aminotransferase, U/L	22.0 (19.0–28.0)	34.0 (23.0–56.0)	< 0.001*
Alanine aminotransferase, U/L	22.0 (16.0–33.0)	33.0 (20.0–64.5)	< 0.001*
Platelet count, ×10^3^/μL	223.0 (186.0–263.0)	151.0 (110.0–204.0)	< 0.001*
Albumin, g/dL	4.6 (4.4–4.8)	4.3 (3.8–4.6)	< 0.001*
Globulin, g/dL	2.7 (2.5–3.0)	2.9 (2.6–3.3)	< 0.001*
Glucose, mg/dL	97.0 (89.0–110.0)	115.0 (99.0–136.0)	< 0.001*
HbA1c, %	5.6 (5.4–6.0)	6.0 (5.4–6.7)	< 0.001*
Total cholesterol, mg/dL	179.0 (154.0–202.5)	160.0 (132.0–189.0)	< 0.001*
Triglycerides, mg/dL	95.0 (68.0–134.0)	107.0 (80.0–142.0)	0.004*
HDL cholesterol, mg/dL	56.0 (46.0–69.0)	49.0 (39.0–63.0)	< 0.001*
eGFR, mL/min/1.73 m^2^	91.0 (79.0–106.0)	86.0 (69.0–106.0)	0.003*

*Note:* All continuous variables exhibited non‐normal distributions according to the Shapiro–Wilk test and are presented as medians (interquartile ranges). Categorical variables are presented as *n* (%). Between‐group comparisons were conducted using the Mann–Whitney *U* test for continuous variables and Pearson's chi‐square test or Fisher's exact test, as appropriate, for categorical variables.

Abbreviations: BMI, body mass index; eGFR, estimated glomerular filtration rate; HbA1c, glycated hemoglobin; HDL, high‐density lipoprotein; HIV, human immunodeficiency virus; MASLD, metabolic dysfunction‐associated steatotic liver disease.

^a^ Cardiometabolic risk factors included overweight or obesity (BMI ≥ 23 kg/m^2^), increased waist circumference (≥ 90 cm in men and ≥ 80 cm in women), diabetes mellitus, hypertension, hypertriglyceridemia (triglycerides ≥ 150 mg/dL), or reduced HDL cholesterol (< 40 mg/dL in men and < 50 mg/dL in women). Dyslipidemia was defined as hypertriglyceridemia or reduced HDL cholesterol.

* Significance was defined as *p* < 0.05.

Our cohort included non–mutually exclusive subgroups comprising 1070 patients with MASLD, 1459 with HBV infection, 250 patients with HCV infection, and 16 patients with documented alcohol‐associated liver disease. Among the 16 patients with alcohol‐associated liver disease, 10 (62.5%) also met the operational criteria for MASLD. Among the 1459 patients with HBV infection, 371 (25.4%) had undergone antiviral therapy for at least 1 year and achieved virologic suppression, whereas 1088 (74.6%) were asymptomatic carriers with no documented history of antiviral therapy and therefore classified as treatment‐naïve. Among the 250 patients with HCV infection, 249 had received antiviral treatment and achieved sustained virologic response, whereas one had persistent detectable HCV RNA at the 1‐year follow‐up and was classified as having treatment failure.

Patients with advanced fibrosis had a significantly higher prevalence of diabetes mellitus (36.2% vs. 10.6%), hypertension (42.7% vs. 23.7%), and alcohol consumption (4.9% vs. 0.2%) compared with those with advanced fibrosis (all *p* < 0.001). MASLD was also more prevalent among patients with advanced fibrosis (53.3% vs. 44.5%, *p* = 0.009). HBV and HCV infections were more common in the advanced fibrosis group than in the nonadvanced fibrosis group (both *p* < 0.01). Human immunodeficiency virus infection was rare, with no significant difference between groups. Laboratory parameters also differed in accordance with fibrosis severity. Patients with advanced fibrosis had significantly higher aspartate aminotransferase and alanine aminotransferase (ALT) levels, lower platelet counts, and lower serum albumin concentrations compared with those without advanced fibrosis (all *p* < 0.01). Median serum globulin levels were also significantly higher in the advanced fibrosis group than in the nonadvanced fibrosis group.

### 3.2 Performance of the Original MAF‐5 Risk Categories

When patients were classified in accordance with the original MAF‐5 risk categories, the prevalence of advanced fibrosis increased in a stepwise manner from 4.5% in the low‐risk group (< 0), to 13.1% in the intermediate‐risk group (0–1), and 37.5% in the high‐risk group (≥ 1) (Table S3). For elastography‐defined advanced fibrosis, the high‐risk category had a sensitivity of 55.3%, a specificity of 89.24%, a positive predictive value of 37.47%, a negative predictive value of 94.48%, and an accuracy of 85.69% (Table S4).

### 3.3 Performance of Serum‐Based Fibrosis Scoring Systems

Table 3 presents a summary of the performance of serum‐based fibrosis scoring systems in distinguishing advanced from nonadvanced liver fibrosis in the overall cohort and in sex‐stratified analysis. In the overall cohort, the scores for all three systems were significantly more favorable in the advanced fibrosis group than in the nonadvanced fibrosis group (all *p* < 0.001). The mean differences between groups were significant for all three scoring systems. MAF‐5 demonstrated the largest mean difference (3.075, 95% CI, 2.725–3.425), followed by FIB‐4 and APRI. The AUROCs were comparable across all scoring systems (Figure S1A), with values ranging from 0.827 for FIB‐4 (95% CI, 0.795–0.858) to 0.839 for MAF‐5 (95% CI: 0.811–0.867). Global model fit, evaluated using pooled Wald *χ* ^2^ statistics across multiple imputed datasets, was statistically significant for all models (all *p* < 0.001).

**TABLE 3:** Performance of serum‐based scoring systems.

Scoring systems	Nonadvanced liver fibrosis (F0–F2)	Advanced liver fibrosis (F3–F4)	Mean difference in scores (95% CI)	*p*	AUROC (95% CI)	Global model tests χ^2^(df)^a^	*p* (model fit)
Overall population (*N* = 2355)							
FIB‐4	1.364 ± 0.8880	3.955 ± 5.032	2.591 (1.679–3.503)	< 0.001*	0.827 (0.795–0.858)	144.9, df = 1	< 0.001*
APRI	0.322 ± 0.334	1.222 ± 2.179	0.899 (0.557–1.242)	< 0.001*	0.832 (0.801–0.863)	103.3, df = 1	< 0.001*
MAF‐5	−1.222 ± 1.840	1.854 ± 2.702	3.075 (2.725–3.425)	< 0.001*	0.839 (0.811–0.867)	271.0, df = 1	< 0.001*
Male sex (*N* = 1135)							
FIB‐4	1.345 ± 0.872	3.552 ± 3.602	2.207 (1.618–2.797)	< 0.001*	0.800 (0.757–0.843)	79.9, df = 1	< 0.001*
APRI	0.345 ± 0.341	1.312 ± 2.305	0.967 (0.592–1.342)	< 0.001*	0.799 (0.752–0.845)	63.9, df = 1	< 0.001*
MAF‐5	−0.669 ± 1.788	2.096 ± 2.879	2.765 (2.282–3.248)	< 0.001*	0.798 (0.753–0.843)	132.9, df = 1	< 0.001*
Female sex (*N* = 1220)							
FIB‐4	1.380 ± 0.883	4.546 ± 6.347	3.165 (1.093–5.238)	0.003*	0.870 (0.828–0.912)	65.7, df = 1	< 0.001*
APRI	0.302 ± 0.326	1.090 ± 1.750	0.787 (0.146–1.429)	0.016*	0.872 (0.833–0.910)	41.3, df = 1	< 0.001*
MAF‐5	−1.711 ± 1.744	1.497 ± 2.388	3.208 (2.721–3.696)	< 0.001*	0.885 (0.854–0.916)	130.6, df = 1	< 0.001*

Abbreviations: APRI, aspartate aminotransferase‐to‐platelet ratio index; AUROC, area under the receiver operating characteristic curve; CI, confidence interval; FIB‐4, Fibrosis‐4 index; MAF‐5; metabolic dysfunction–associated fibrosis‐5 score.

^a^ Global model performance was evaluated using Wald *χ* ^2^ tests pooled across multiple imputed datasets.

* Significance was defined as *p* < 0.05.

### 3.4 Sex‐Stratified Analysis

Sex‐stratified analysis was presented as follows (Table 3). Among men, all scores were significantly more favorable in the advanced fibrosis group than in the nonadvanced fibrosis group (all *p* < 0.001), with AUROCs ranging from 0.798 to 0.800 (Figure S1B,C). For women, the scoring systems exhibited modestly higher discrimination overall, with AUROCs ranging from 0.870 to 0.885. Global model tests indicated an adequate model fit for all scoring systems in both men and women.

### 3.5 Performance in Etiology‐Specific Subgroups

Table 4 presents the performance of the serum‐based fibrosis scoring systems in the MASLD (*n* = 1070), HBV infection (*n* = 1459), and HCV infection (*n* = 250) subgroups. In each subgroup, the mean scores for all scoring systems were significantly higher in the advanced fibrosis group than in the nonadvanced fibrosis (all *p* < 0.05). In the MASLD subgroup, the AUROCs ranged from 0.806 to 0.822 (Figure S1D–F). In the HBV infection subgroup, the AUROCs were also similar across the three scoring systems, ranging from 0.830 to 0.843. In the HCV infection subgroup, APRI demonstrated the highest discrimination (AUROC: 0.822, 95% CI, 0.749–0.896), followed by MAF‐5 (AUROC, 0.810; 95% CI, 0.735–0.886), and FIB‐4 (AUROC, 0.777; 95% CI, 0.695–0.859). Furthermore, an MAF‐5 score ≥ 1 demonstrated a sensitivity, specificity, and accuracy of 64.9%, 84.8%, and 79.7%, respectively, in the MASLD subgroup; 52.0%, 90.5%, and 87.3%, respectively, in the HBV infection subgroup; and 41.0%, 91.5%, and 83.6%, respectively, in the HCV infection subgroup (Table S4).

**TABLE 4:** Model performance of FIB‐4, APRI, and MAF‐5 in MASLD, HBV infection, and HCV infection subgroups.

Scoring systems	Nonadvanced liver fibrosis (F0–F2)	Advanced liver fibrosis (F3–F4)	Mean difference in scores (95% CI)	*p*	AUROC curve (95% CI)	Global model tests χ^2^(df)^a^	*p* (model fit)
MASLD subgroup (*N* = 1070)							
FIB‐4	1.240 ± 0.780	3.123 ± 3.099	1.883 (1.360–2.406)	< 0.001*	0.806 (0.763–0.850)	75.5, df = 1	< 0.001*
APRI	0.316 ± 0.254	0.906 ± 1.174	0.590 (0.392–0.787)	< 0.001*	0.819 (0.776–0.863)	72.0, df = 1	< 0.001*
MAF‐5	−0.669 ± 1.882	2.178 ± 2.561	2.847 (2.399–3.296)	< 0.001*	0.822 (0.781–0.863)	136.8, df = 1	< 0.001*
Hepatitis B infection subgroup (*N* = 1459)							
FIB‐4	1.394 ± 0.856	3.824 ± 4.659	2.429 (1.603–3.255)	< 0.001*	0.833 (0.790–0.875)	73.0, df = 1	< 0.001*
APRI	0.331 ± 0.347	1.249 ± 2.226	0.918 (0.524–1.312)	< 0.001*	0.830 (0.785–0.875)	55.2, df = 1	< 0.001*
MAF‐5	−1.303 ± 1.375	1.757 ± 2.791	3.060 (2.556–3.564)	< 0.001*	0.843 (0.804–0.882)	150.0, df = 1	< 0.001*
Hepatitis C infection subgroup (*N* = 250)							
FIB‐4	1.687 ± 0.990	4.031 ± 4.459	2.344 (0.900–3.788)	0.004*	0.777 (0.695–0.859)	18.2, df = 1	< 0.001*
APRI	0.289 ± 0.163	0.916 ± 1.708	0.628 (0.087–1.168)	0.023*	0.822 (0.749–0.896)	15.7, df = 1	< 0.001*
MAF‐5	−1.438 ± 1.592	0.687 ± 1.995	2.124 (1.445–2.804)	< 0.001*	0.810 (0.735–0.886)	31.6, df = 1	< 0.001*

Abbreviations: APRI, aspartate aminotransferase‐to‐platelet ratio index; AUROC, area under the receiver operating characteristic curve; CI, confidence interval; FIB‐4, Fibrosis‐4 index; MAF‐5; metabolic dysfunction–associated fibrosis‐5 score; MASLD, metabolic dysfunction‐associated steatotic liver disease.

^a^ Global model performance was evaluated using Wald *χ* ^2^ tests pooled across multiple imputed datasets.

* Significance was defined as *p* < 0.05.

### 3.6 Model Performance Comparisons

Table 5 presents a summary of the pooled cfNRI values for alternative fibrosis scores compared with FIB‐4 in the overall cohort and pre‐specified subgroups. In the overall cohort, MAF‐5 demonstrated a significantly positive cfNRI value (0.168; 95% CI, 0.093–0.244; *p* < 0.001), whereas APRI demonstrated a significantly negative cfNRI value (−0.663; 95% CI, −0.724 to −0.603; *p* < 0.001). MAF‐5 also demonstrated significantly positive cfNRI values in the HBV infection (0.239, 95% CI 0.131–0.347; *p* < 0.001) and HCV infection (0.241, 95% CI, 0.166–0.317; *p* < 0.001) subgroups. In the MASLD subgroup, the cfNRI value was positive but did not reach statistical significance (0.196, 95% CI, −0.002 to 0.393; *p* = 0.052). APRI demonstrated negative cfNRI values in the overall cohort and in the sex‐stratified, MASLD, and HBV infection subgroups, whereas its cfNRI in the HCV subgroup was not statistically significant. In the sex‐stratified analyses, MAF‐5 demonstrated a significantly positive cfNRI value among men (0.153, 95% CI, 0.085–0.220; *p* < 0.001) but not among women (0.038; 95% CI, −0.158 to 0.234; *p* = 0.697).

**TABLE 5:** Head‐to‐head cfNRI comparisons with FIB‐4 in the overall cohort and etiology‐specific subgroups.

Scoring systems	Pooled cfNRI (95% CI)	*p*
Overall population (*N* = 2355)		
FIB‐4	Reference	—
APRI	−0.663 (−0.724, −0.603)	< 0.001*
MAF‐5	0.168 (0.093, 0.2440)	< 0.001*
Male sex (*N* = 1135)		
FIB‐4	Reference	—
APRI	−0.505 (−0.581, −0.428)	< 0.001*
MAF‐5	0.153 (0.085, 0.220)	< 0.001*
Female sex (*N* = 1220)		
FIB‐4	Reference	—
APRI	−0.998 (−1.067, −0.929)	< 0.001*
MAF‐5	0.038 (−0.158, 0.234)	0.697
Subgroup of MASLD (*N* = 1070)		
FIB‐4	Reference	—
APRI	−0.447 (−0.672, −0.222)	< 0.001*
MAF‐5	0.196 (−0.002, 0.393)	0.052
Subgroup of hepatitis B infection (*N* = 1459)		
FIB‐4	Reference	—
APRI	−0.678 (−0.767, −0.588)	< 0.001*
MAF‐5	0.239 (0.131, 0.347)	< 0.001*
Subgroup of hepatitis C infection (*N* = 250)		
FIB‐4	Reference	—
APRI	0.197 (−0.282, 0.675)	0.413
MAF‐5	0.241 (0.166, 0.317)	< 0.001*

*Note:* Estimates were calculated within each of 50 imputed datasets and pooled using Rubin's rules.

Abbreviations: APRI, aspartate aminotransferase‐to‐platelet ratio index; cfNRI, category‐free net reclassification improvement; FIB‐4, Fibrosis‐4 index; MAF‐5; metabolic dysfunction–associated fibrosis‐5 score; MASLD, metabolic dysfunction‐associated steatotic liver disease.

* Significance was defined as *p* < 0.05.

### 3.7 Sensitivity Analysis I

The complete‐case analysis included 2111 out of 2355 patients (89.64%), including 1004 men, 1107 women, 959 patients with MASLD, 1304 patients with HBV infection, and 241 patients with HCV infection. All three fibrosis scores remained significantly higher in the advanced fibrosis group than in the nonadvanced fibrosis group in the overall cohort and all prespecified subgroups (Table S5). In the overall cohort, the AUROCs were 0.810 for FIB‐4, 0.821 for APRI, and 0.804 for MAF‐5. Etiology‐specific AUROCs ranged from 0.785 to 0.798 in MASLD, 0.799 to 0.801 in HBV infection, and 0.759 to 0.833 in HCV infection. In terms of model comparison, APRI demonstrated less favorable reclassification compared with FIB‐4 in the overall cohort and in the male, female, MASLD, and HBV infection subgroups but more favorable reclassification in the HCV infection subgroup. MAF‐5 did not significantly improve reclassification compared with FIB‐4 in the overall cohort or in the male, MASLD, HBV infection, or HCV infection subgroups, and it exhibited less favorable reclassification among women (Table S6).

### 3.8 Sensitivity Analysis II

Among patients with MASLD without coexisting HBV or HCV infections, the AUROCs remained comparable across FIB‐4, APRI, and MAF‐5 (0.835, 0.820, and 0.827, respectively; Table S5). APRI exhibited a negative cfNRI value, whereas the cfNRI for MAF‐5 did not significantly differ from zero (Table S6).

## 4 Discussion

When evaluated against VCTE‐defined advanced fibrosis, the three serum‐based fibrosis scores exhibited comparable discrimination, with AUROCs ranging from 0.82 to 0.88 in the overall cohort. Although overall discrimination was similar, reclassification analyses revealed differences in predictive performance relative to FIB‐4. These findings indicate that similar AUROCs do not necessarily translate into equivalent reclassification performance. Accordingly, both discrimination and reclassification should be considered when comparing serum‐based fibrosis scores.

Fibrosis severity was classified using liver stiffness measured by VCTE, a well‐validated, noninvasive, and scalable method for fibrosis assessment, particularly when liver biopsy is impractical or not clinically indicated [8, 24]. Our classification of advanced fibrosis by using thresholds corresponding to F3–F4 is clinically meaningful because it identifies patients who are at a substantially increased risk of cirrhosis‐related complications, hepatocellular carcinoma, and liver‐related mortality [7, 25]. To account for etiology‐specific differences in the relationship between liver stiffness and fibrosis, etiology‐specific liver stiffness thresholds, including ALT‐stratified thresholds for HBV infection, were applied in accordance with previous validation studies and routine clinical practice, thereby strengthening the clinical relevance and interpretability of the outcome definition [17, 26].

Our primary reclassification analysis suggested an advantage of MAF‐5 beyond conventional disclination metrics. Compared with FIB‐4, MAF‐5 was associated with significantly greater individual risk classification over FIB‐4 in the overall cohort (cfNRI, 0.168; 95% CI, 0.093–0.244) and in the HBV infection (0.239; 95% CI, 0.131–0.347) and HCV infection (0.241; 95% CI, 0.166–0.317) subgroups. By contrast, APRI generally yielded negative cfNRI estimates, except in the HCV infection subgroup, in which its cfNRI estimate was positive but not statistically significant. These findings should also be interpreted in the context of the populations in which the fibrosis scores were originally developed. FIB‐4 was initially developed for patients with HCV and human immunodeficiency virus coinfection, APRI was developed for patients with HCV infection, and MAF‐5 was developed for a general population with metabolic dysfunction [7, 10, 12, 27]. Although these scores have subsequently been evaluated in other liver diseases, their comparative assessment in an East Asian clinical cohort with a substantial burden of HBV and HCV infection provides useful evidence regarding their external performance in this population [28, 29]. Collectively, our findings address to calls for further evaluation of noninvasive fibrosis scores in Asian populations and patients with viral hepatitis, [30, 31] while supporting the consideration of liver disease etiology when interpreting these scores in clinical practice.

Although MAF‐5 was developed in a general population with metabolic dysfunction, [12] it combines aspartate aminotransferase level and platelet count, which reflect liver injury and fibrosis‐associated portal hypertension. In patients with HBV infection, waist circumference, body mass index, and diabetes capture metabolic stress relevant to fibrosis progression [32, 33, 34]. In patients with HCV infection, insulin resistance and cardiometabolic risk factors are linked to fibrosis progression [35, 36]. In the present study, MAF‐5 exhibited broadly similar discrimination in the MASLD, HBV infection, and HCV infection subgroups, with AUROCs of 0.839, 0.843, and 0.810, respectively. MAF‐5 also demonstrated similar discrimination in the broader MASLD subgroup, which included patients with coexisting HBV or HCV infection, and the MASLD subgroup without HBV or HCV infection (AUROC, 0.839 vs. 0.827). This finding suggests that the inclusion of patients with coexisting viral hepatitis did not materially affect the discriminatory performance of MAF‐5 in the MASLD subgroup. Furthermore, the prevalence of advanced fibrosis increased in a stepwise manner across the original MAF‐5 risk categories. The MAF‐5 ≥ 1 category exhibited high specificity and a negative predictive value for advanced fibrosis in the MASLD and HBV infection subgroups.

On the other hand, MAF‐5 did not improve reclassification over FIB‐4 among women, despite exhibiting satisfactory discrimination. Because MAF‐5 incorporates anthropometric and metabolic variables, previous evidence indicating stronger associations of waist circumference and visceral obesity with fibrosis in Chinese men provides a potential explanation for the greater incremental contribution of MAF‐5 in men [37]. However, the reclassification advantage observed among men was attenuated in the complete‐case analysis; therefore, our sex‐stratified cfNRI findings should be interpreted with caution. In addition, the AUROCs from the complete‐case analysis remained comparable to those from the multiply imputed analysis, mitigating concerns that missing platelet measurements affected the discrimination findings.

This study has several strengths that support the validity of its findings. First, the fibrosis scores were recalculated using standardized methods within a single cohort, minimizing methodological heterogeneity across the scoring systems. Second, etiology‐specific FibroScan thresholds were applied, enabling a more accurate classification of fibrosis severity across different liver disease etiologies. Third, the results were consistent across sex‐ and etiology‐stratified analyses, supporting their robustness. Finally, multiple imputation minimized potential bias associated with missing data and enabled the inclusion of a large real‐world population undergoing elastography.

Despite these strengths, this study has several limitations that must be acknowledged. First, this was a retrospective single‐center study conducted in an East Asian clinical setting. Therefore, residual confounding and measurement variability inherent to routine clinical care cannot be excluded. Second, the cohort included patients with overlapping liver disease etiologies. Because MAF‐5 was originally developed for a population with metabolic dysfunction, [12] our analysis across different liver etiologies requires cautious interpretation and further validation. Although antiviral treatment status and categorical virologic responses were available, quantitative HBV DNA and HCV RNA levels measured contemporaneously with VCTE were unavailable. Therefore, the effect of viral load on liver stiffness and fibrosis score performance could not be evaluated. Third, VCTE‐derived liver stiffness rather than liver biopsy was used to define advanced fibrosis. Therefore, our findings indicate the performance of the fibrosis scores in identifying VCTE‐defined advanced fibrosis and should not be interpreted as validation against histologic fibrosis staging. However, the American Association for the Study of Liver guidelines incorporate both imaging‐ and blood‐based noninvasive tests into fibrosis assessment pathways [16, 38]. Finally, our relatively small HCV subgroup limited the precision of the HCV‐specific estimates, and the small number of patients with alcohol‐associated liver disease (*n* = 16) precluded a specific evaluation of this subgroup. Larger external cohorts are required to validate the performance of serum‐based scores in these subgroups.

From a clinical perspective, our findings suggest that when serum‐based fibrosis scores exhibit similar overall discrimination, the selection of a score should also consider reclassification performance, interpretability, and operational feasibility. MAF‐5 can provide incremental value for individual‐level risk stratification to guide further diagnostic evaluation or specialist referral. In addition, MAF‐5 demonstrated a strong discriminatory ability in identifying VCTE‐defined advanced fibrosis (F3–F4) in patients with MASLD, HBV infection, and HCV infection. These findings extend the evaluation of MAF‐5 to an East Asian clinical setting that includes patients with diverse liver disease etiologies.

## Funding

This study was financially supported of the Higher Education Sprout Project of the Ministry of Education (MOE) in Taiwan (grant number: DP2‐TMU‐114‐HIS‐01). This study was also supported by Taipei Medical University Hospital, Taipei, Taiwan (grant number: 115TMU‐TMUH‐22) and Taipei Medical University.

## Ethics Statement

The study protocol was approved by the Joint Institutional Review Board of Taipei Medical University, which waived the requirement for informed consent because of the retrospective design and use of de‐identified data (approval number: N202509023).

## Conflicts of Interest

The authors declare no conflicts of interest.

## Supporting information


**Figure S1:** Areas under the receiver operating characteristic curves (AUROCs) are presented for (A) the overall population, (B) male participants, (C) female participants, and the subgroups with (D) metabolic dysfunction‐associated steatotic liver disease (MASLD), (E) hepatitis B virus (HBV) infection, and (F) hepatitis C virus (HCV) infection. Predicted probabilities were estimated separately in each of the 50 multiply imputed datasets and averaged across imputations to generate the AUROC estimates.

**Table S1:** Etiology‐specific VCTE‐derived liver stiffness thresholds used to classify fibrosis severity.
**Table S2:** Missing data summary.
**Table S3:** Advanced fibrosis prevalence by original MAF‐5 categories.
**Table S4:** Diagnostic performance of original MAF‐5 categories by subgroup.
**Table S5:** Model performance of FIB‐4, APRI, and MAF‐5 in complete‐case and isolated MASLD sensitivity analyses.
**Table S6:** Head‐to‐head cfNRI comparisons with FIB‐4 in complete‐case and isolated MASLD sensitivity analysis.

## Data Availability

The data that support the findings of this study are available on request from the corresponding author. The data are not publicly available due to privacy or ethical restrictions.
