# Supplementary material for: Diagnostic Accuracy of Serum‐Based Fibrosis Scores for Clinically Significant Liver Fibrosis in an East Asian Cohort
Source: Kaohsiung J Med Sci. 2026 Sep 24:e70296. Online ahead of print. doi: 10.1002/kjm2.70296 (PMC13602177; doi:10.1002/kjm2.70296)
Supplement: Supplementary file 1 [file KJM2-9999-e70296-s001.docx]

Supplemental Materials.

Supplemental Table S1. Etiology-specific VCTE-derived liver stiffness thresholds used to classify fibrosis severity

Supplemental Table S2. Missing data summary

Supplemental Table S3 Advanced fibrosis prevalence by original MAF-5 categories

Supplemental Table S4. Diagnostic performance of original MAF-5 categories by subgroup

Supplemental Table S5. Model performance of FIB-4, APRI, and MAF-5 in complete-case and isolated MASLD sensitivity analyses

Supplemental Table S6. Head-to-head cfNRI comparisons with FIB-4 in complete-case and isolated MASLD sensitivity analysis

Supplemental Table S1. Etiology-specific VCTE-derived liver stiffness thresholds used to classify fibrosis severity

| Definition | Operational criteria | VCTE-based staging | Reference |
| --- | --- | --- | --- |
| General chronic liver disease / unspecified etiology | Patients without a specific HBV, HCV infection, or alcohol-associated liver disease threshold. | F0: LSM <7.0 kPa F1-F2: LSM 7.0–9.4 kPa F3: LSM 9.5–12.4 kPa F4: LSM ≥12.5 kPa | AASLD Practice Guideline[1] |
| MASLD | CAP ≥248 dB/m plus ≥1 cardiometabolic risk factor. Coexisting HBV, HCV, or alcohol-associated liver disease was permitted; therefore, this subgroup was non-mutually exclusive. | No coexisting HBV, HCV, or alcohol-associated liver disease: general thresholds. Overlapping liver disease: corresponding etiology-specific LSM threshold. | AASLD Practice Guideline[1];  Karlas et al.[2]  Rinella et al.[3];; Tilg et al.[4] |
| HBV infection with normal alanine aminotransferase | Recorded HBV diagnosis with normal ALT. | F0: LSM <6.0 kPa F1-F2: LSM 6.0–8.9 kPa F3: LSM 9.0–11.9 kPa F4: LSM ≥12.0 kPa | Karlas et al. [5] |
| HBV infection with elevated alanine aminotransferase | Recorded HBV diagnosis with elevated ALT. | F0: LSM <7.5 kPa F1-F2: LSM 7.5-11.9 kPa F3: LSM 12.0-13.3 kPa F4: LSM ≥13.4 kPa. | Karlas et al. [5] |
| HCV infection | Recorded HCV diagnosis. | F0: LSM <6.0 kPa F1-F2: LSM 6.0–8.9 kPa F3: LSM 9.0–11.9 kPa F4: LSM ≥12.0 kPa | Arena et al.[6] |
| Alcohol-associated liver disease | Recorded alcohol-associated liver disease | F0-F2: LSM <9.5 kPa F3: LSM 9.5–12.4 kPa F4: LSM ≥12.5 kPa | AASLD Practice Guideline[1]; Pavlov et al.[7] |
| MASLD without HBV or HCV infection  (sensitivity analysis) | CAP ≥248 dB/m plus ≥1 cardiometabolic risk factor, excluding patients with recorded HBV, HCV, or alcohol-associated liver disease. | General chronic liver disease thresholds. | AASLD Practice Guideline[1];  Karlas et al.[2];  Rinella et al.[3]; Tilg et al.[4] |

CAP, controlled attenuation parameter; F0–F4, fibrosis stages; HBV, hepatitis B virus; HCV, hepatitis C virus; LSM, liver stiffness measurement; MASLD, metabolic dysfunction-associated steatotic liver disease; VCTE, vibration controlled transient elastography. Cardiometabolic risk factors included overweight or obesity, increased waist circumference, diabetes mellitus, hypertension, hypertriglyceridemia, or reduced high-density lipoprotein cholesterol. Advanced fibrosis was defined as F3 or F4.

Cardiometabolic risk factors included overweight or obesity (BMI ≥23 kg/m²), increased waist circumference (≥90 cm in men and ≥80 cm in women), diabetes mellitus, hypertension, hypertriglyceridemia (triglycerides ≥150 mg/dL), or reduced HDL cholesterol (<40 mg/dL in men and <50 mg/dL in women).

Reference:

1.	Sterling RK, Duarte-Rojo A, Patel K, Asrani SK, Alsawas M, Dranoff JA, et al. AASLD Practice Guideline on imaging-based noninvasive liver disease assessment of hepatic fibrosis and steatosis. Hepatology. 2025;81(2):672-724.

2.	Karlas T, Petroff D, Sasso M, Fan JG, Mi YQ, de Lédinghen V, et al. Individual patient data meta-analysis of controlled attenuation parameter (CAP) technology for assessing steatosis. J Hepatol. 2017;66(5):1022-30.

3.	Rinella ME, Lazarus JV, Ratziu V, Francque SM, Sanyal AJ, Kanwal F, et al. A multisociety Delphi consensus statement on new fatty liver disease nomenclature. J Hepatol. 2023;79(6):1542-56.

4.	Tilg H, Petta S, Stefan N, Targher G. Metabolic Dysfunction–Associated Steatotic Liver Disease in Adults: A Review. JAMA. 2026;335(2):163-74.

5.	Karlas T, Petroff D, Sasso M, Fan JG, Mi YQ, de Lédinghen V, et al. Impact of controlled attenuation parameter on detecting fibrosis using liver stiffness measurement. Alimentary Pharmacology & Therapeutics. 2018;47(7):989-1000.

6.	Arena U, Vizzutti F, Abraldes JG, Corti G, Stasi C, Moscarella S, et al. Reliability of transient elastography for the diagnosis of advanced fibrosis in chronic hepatitis C. Gut. 2008;57(9):1288-93.

7.	Pavlov CS, Casazza G, Nikolova D, Tsochatzis E, Burroughs AK, Ivashkin VT, et al. Transient elastography for diagnosis of stages of hepatic fibrosis and cirrhosis in people with alcoholic liver disease. Cochrane Database Syst Rev. 2015;1(1):Cd010542.

Supplemental Table S2. Missing data summary

| Variables | Available data, n | Missing, n (%) |
| --- | --- | --- |
| Age | 2355 | 0 (0.0%) |
| Sex | 2355 | 0 (0.0%) |
| BMI | 2351 | 4 (0.17%) |
| Waist circumference | 2337 | 18 (0.76%) |
| AST | 2224 | 131 (5.56%) |
| ALT | 2307 | 48 (2.03%) |
| Platelet | 2112 | 243 (10.36%) |
| Controlled attenuation parameter | 2355 | 0 (0.0%) |
| Liver stiffness measurement | 2355 | 0 (0.0%) |
| Albumin | 1398 | 957 (40.64%) |
| Globulin | 789 | 1566 (66.50%) |
| Glucose | 1785 | 570 (24.20%) |
| HbA1c | 1438 | 917 (38.94%) |
| Total cholesterol | 1833 | 522 (22.17%) |
| Triglycerides | 1836 | 519 (22.04%) |
| HDL cholesterol | 1525 | 830 (35.24%) |
| eGFR | 2225 | 130 (5.52%) |

Abbreviations: ALT, alanine aminotransferase; AST, aspartate aminotransferase; BMI, body mass index. Missing values were handled using multiple imputation (50 imputations).

Supplemental Table S3. Advanced fibrosis prevalence by original MAF-5 categories

| MAF-5 category | N | No advanced fibrosis (F0–F2), N | Advanced fibrosis (F3–F4), N | Advanced fibrosis (F3–F4), % |
| --- | --- | --- | --- | --- |
| Overall (N=2,355) | |  |  |  |
| <0 | 1,747 | 1,669 | 78 | 4.5% |
| 0–1 | 245 | 213 | 32 | 13.1% |
| >1 | 363 | 227 | 136 | 37.5% |
| MASLD (N=1,070) | |  |  |  |
| <0 | 671 | 637 | 34 | 5.1% |
| 0–1 | 143 | 131 | 12 | 8.4% |
| >1 | 256 | 171 | 85 | 33.2% |
| HBV (N=1,459) | |  |  |  |
| <0 | 1,119 | 1,080 | 39 | 3.5% |
| 0–1 | 149 | 129 | 20 | 13.4% |
| >1 | 191 | 127 | 64 | 33.5% |
| HCV (N=250) | |  |  |  |
| <0 | 190 | 174 | 16 | 8.4% |
| 0–1 | 26 | 19 | 7 | 26.9% |
| >1 | 34 | 18 | 16 | 47.1% |

Abbreviations: HBV, hepatitis B virus; HCV, hepatitis C virus; MAF-5, metabolic dysfunction-associated fibrosis-5; N, number.

Supplemental Table S4. Diagnostic performance of original MAF-5 categories by subgroup

| Subgroup | MAF-5 category as test-positive | Advanced fibrosis, n (%) | TP | FP | TN | FN | Sensitivity | Specificity | PPV | NPV | Accuracy |
| --- | --- | --- | --- | --- | --- | --- | --- | --- | --- | --- | --- |
| Overall | MAF-5 <0 | 78 (4.5%) | 78 | 1,669 | 440 | 168 | 31.71% | 20.86% | 4.46% | 72.37% | 22.00% |
| (N=2,355) | MAF-5 0–1 | 32 (13.1%) | 32 | 213 | 1,896 | 214 | 13.01% | 89.90% | 13.06% | 89.86% | 81.87% |
|  | MAF-5 ≥1 | 136 (37.5%) | 136 | 227 | 1,882 | 110 | 55.28% | 89.24% | 37.47% | 94.48% | 85.69% |
| MASLD | MAF-5 <0 | 34 (4.9%) | 34 | 637 | 302 | 970 | 25.95% | 32.16% | 5.07% | 75.69% | 31.40% |
| (N=1,070) | MAF-5 0–1 | 12 (8.2%) | 12 | 131 | 808 | 119 | 9.16% | 86.05% | 8.39% | 87.16% | 76.64% |
|  | MAF-5 ≥1 | 85 (33.2%) | 85 | 171 | 768 | 46 | 64.89% | 81.79% | 33.20% | 94.35% | 79.72% |
| HBV | MAF-5 <0 | 39 (3.5%) | 39 | 1,080 | 256 | 84 | 31.71% | 19.16% | 3.49% | 75.29% | 20.22% |
| (N=1,459) | MAF-5 0–1 | 20 (13.4%) | 20 | 129 | 1,207 | 103 | 16.26% | 90.34% | 13.42% | 92.14% | 84.10% |
|  | MAF-5 ≥1 | 64 (33.5%) | 64 | 127 | 1,209 | 59 | 52.03% | 90.49% | 33.51% | 95.35% | 87.25% |
| HCV | MAF-5 <0 | 16 (8.4%) | 16 | 174 | 37 | 23 | 41.03% | 17.54% | 8.42% | 61.67% | 21.20% |
| (N=250) | MAF-5 0–1 | 7 (26.9%) | 7 | 19 | 192 | 32 | 17.95% | 91.00% | 26.92% | 85.71% | 79.60% |
|  | MAF-5 ≥1 | 16 (47.1%) | 16 | 18 | 193 | 23 | 41.03% | 91.47% | 47.06% | 89.35% | 83.60% |

Note: For each row, test-positive indicates membership in the specified MAF-5 category; all other MAF-5 categories were treated as test-negative. Advanced fibrosis was defined as FibroScan-based F3 or F4. MASLD-compatible status was defined as CAP ≥230 dB/m plus at least one cardiometabolic risk factor. FN, false negative; FP, false positive; HBV, hepatitis B virus; HCV, hepatitis C virus; MAF-5, metabolic dysfunction-associated fibrosis-5 score; MASLD, metabolic dysfunction-associated steatotic liver disease; NPV, negative predictive value; PPV, positive predictive value; TN, true negative; TP, true positive.

Supplemental Table S5. Model performance of FIB-4, APRI, and MAF-5 in complete-case and isolated MASLD sensitivity analyses

| Scoring Systems | Non-advanced liver fibrosis (F0–F2) | Advanced liver fibrosis (F3–F4) | Mean Difference of Scores (95% CI) | *P* value | AUROC (95% CI) | Global model 𝜒^2^(*df*)† | *P* value for model fit |
| --- | --- | --- | --- | --- | --- | --- | --- |
| Sensitivity Analysis I: Complete-case cohort | | | |  |  |  |  |
| Overall (*N* = 2,111) | | | |  |  |  |  |
| FIB-4 | 1.312 ± 0.876 | 3.793 ± 4.302 | 2.481 (1.932–3.031) | <0.001* | 0.810 (0.776–0.844) | 344.7 (1) | <0.001* |
| APRI | 0.003 ± 0.003 | 0.012 ± 0.020 | 0.009 (0.006–0.011) | <0.001* | 0.821 (0.788–0.854) | 228.8 (1) | <0.001* |
| MAF-5 | −1.679 ± 2.600 | 1.270 ± 3.360 | 2.949 (2.505–3.393) | <0.001* | 0.804 (0.769–0.838) | 272.5 (1) | <0.001* |
| Male sex (*N* = 1,004) | |  |  |  |  |  |  |
| FIB-4 | 1.299 ± 0.886 | 3.471 ± 3.663 | 2.173 (1.566–2.779) | <0.001* | 0.778 (0.731–0.824) | 158.2 (1) | <0.001* |
| APRI | 0.003 ± 0.003 | 0.013 ± 0.023 | 0.010 (0.006–0.013) | <0.001* | 0.782 (0.736–0.828) | 129.0 (1) | <0.001* |
| MAF-5 | −1.146 ± 2.634 | 1.513 ± 3.560 | 2.659 (2.046–3.271) | <0.001* | 0.762 (0.715–0.810) | 121.6 (1) | <0.001* |
| Female sex (*N* = 1,107) | |  |  |  |  |  |  |
| FIB-4 | 1.323 ± 0.867 | 4.281 ± 5.103 | 2.958 (1.917–3.999) | <0.001* | 0.862 (0.814–0.909) | 193.1 (1) | <0.001* |
| APRI | 0.003 ± 0.003 | 0.010 ± 0.013 | 0.007 (0.005–0.010) | <0.001* | 0.869 (0.823–0.916) | 92.0 (1) | <0.001* |
| MAF-5 | −2.131 ± 2.484 | 0.901 ± 3.013 | 3.032 (2.400–3.665) | <0.001* | 0.849 (0.800–0.899) | 139.0 (1) | <0.001* |
| Subgroup of MASLD (*N* = 959) | |  |  |  |  |  |  |
| FIB-4 | 1.158 ± 0.719 | 2.872 ± 3.066 | 1.714 (1.171–2.256) | <0.001* | 0.785 (0.736–0.834) | 158.1 (1) | <0.001* |
| APRI | 0.003 ± 0.002 | 0.008 ± 0.012 | 0.006 (0.003–0.008) | <0.001* | 0.798 (0.750–0.846) | 119.8 (1) | <0.001* |
| MAF-5 | −1.134 ± 2.718 | 1.629 ± 3.463 | 2.764 (2.126–3.401) | <0.001* | 0.796 (0.748–0.844) | 121.9 (1) | <0.001* |
| Subgroup of Hepatitis B infection (*N* = 1,304) | | |  |  |  |  |  |
| FIB-4 | 1.351 ± 0.844 | 3.688 ± 4.732 | 2.337 (1.488–3.186) | <0.001* | 0.799 (0.751–0.847) | 169.8 (1) | <0.001* |
| APRI | 0.003 ± 0.004 | 0.012 ± 0.022 | 0.009 (0.005–0.013) | <0.001* | 0.799 (0.750–0.847) | 101.0 (1) | <0.001* |
| MAF-5 | −1.670 ± 2.405 | 1.092 ± 3.504 | 2.762 (2.120–3.405) | <0.001* | 0.801 (0.753–0.849) | 137.0 (1) | <0.001* |
| Subgroup of Hepatitis C infection (*N* = 241) | | |  |  |  |  |  |
| FIB-4 | 1.639 ± 0.993 | 3.900 ± 4.508 | 2.261 (0.752–3.770) | 0.004* | 0.759 (0.665–0.853) | 31.6 (1) | <0.001* |
| APRI | 0.003 ± 0.002 | 0.009 ± 0.017 | 0.006 (0.001–0.012) | 0.029* | 0.833 (0.750–0.917) | 40.9 (1) | <0.001* |
| MAF-5 | −1.710 ± 2.205 | 0.654 ± 1.987 | 2.364 (1.640–3.089) | <0.001* | 0.812 (0.725–0.899) | 42.0 (1) | <0.001* |
| Sensitivity Analysis II: Isolated MASLD after exclusion of HBV and HCV (*N* = 369) | | | | |  |  |  |
| FIB-4 | 1.079 ± 0.779 | 3.243 ± 3.343 | 2.163 (1.197–3.129) | <0.001* | 0.835 (0.770–0.901) | 28.5 (1) | <0.001* |
| APRI | 0.327 ± 0.293 | 0.921 ± 0.941 | 0.594 (0.322–0.867) | <0.001* | 0.820 (0.743–0.898) | 27.3 (1) | <0.001* |
| MAF-5 | −0.391 ± 2.125 | 2.677 ± 2.498 | 3.068 (2.301–3.835) | <0.001* | 0.827 (0.757–0.896) | 47.5 (1) | <0.001* |

Values are presented as mean ± SD unless. Complete-case analyses were restricted to patients with nonmissing advanced fibrosis status, platelet count, and all three fibrosis scores. For the complete-case cohort, model fit was assessed using likelihood-ratio χ² tests from logistic regression models. For the isolated MASLD sensitivity analysis based on multiply imputed data, model fit was assessed using Rubin-pooled Wald χ² statistics. AUROC, area under the receiver operating characteristic curve; APRI, aspartate aminotransferase-to-platelet ratio index; cfNRI, category-free net reclassification improvement; FIB-4, fibrosis-4 index; MAF-5, metabolic dysfunction-associated fibrosis-5 score; MASLD, metabolic dysfunction-associated steatotic liver disease. *Significance was defined as P < 0.05.

Supplemental Table S6. Head-to-head cfNRI comparisons with FIB-4 in complete-case and isolated MASLD sensitivity analysis

| Scoring Systems | cfNRI (95% CI) | *P* value |
| --- | --- | --- |
| Sensitivity Analysis I: Complete-case cohort | |  |
| Overall (*N* = 2,111) | |  |
| FIB-4 | Reference | - |
| APRI | −0.629 (−0.761, −0.498) | <0.001* |
| MAF-5 | −0.008 (−0.142, 0.126) | 0.912 |
| Male sex (*N* = 1,004) |  |  |
| FIB-4 | Reference | - |
| APRI | −0.411 (−0.586, −0.237) | <0.001* |
| MAF-5 | 0.028 (−0.149, 0.204) | 0.757 |
| Female sex (*N* = 1,107) | |  |
| FIB-4 | Reference | - |
| APRI | −0.990 (−1.186, −0.795) | <0.001* |
| MAF-5 | −0.307 (−0.508, −0.106) | 0.003* |
| Subgroup of MASLD (*N* = 959) | |  |
| FIB-4 | Reference | - |
| APRI | −0.363 (−0.549, −0.177) | <0.001* |
| MAF-5 | 0.009 (−0.178, 0.197) | 0.922 |
| Subgroup of Hepatitis B infection (*N* = 1,304) | |  |
| FIB-4 | Reference | - |
| APRI | −0.655 (−0.837, −0.473) | <0.001* |
| MAF-5 | 0.045 (−0.141, 0.231) | 0.635 |
| Subgroup of Hepatitis C infection (*N* = 241) | |  |
| FIB-4 | Reference | - |
| APRI | 0.660 (0.321, 0.999) | <0.001* |
| MAF-5 | 0.302 (−0.047, 0.650) | 0.090 |
| Sensitivity Analysis II: MASLD without HBV or HCV infection (*N* = 369) | | |
| FIB-4 | Reference |  |
| APRI | -0.616 (-1.003, -0.230) | 0.002* |
| MAF-5 | 0.239 (-0.091, 0.569) | 0.155 |

Complete-case cfNRI estimates were calculated using observed complete cases. Isolated MASLD cfNRI estimates were calculated within each of 50 imputed datasets and pooled using Rubin’s rules. Positive cfNRI values indicate better reclassification than FIB-4; negative values indicate worse reclassification than FIB-4. APRI, aspartate aminotransferase-to-platelet ratio index; cfNRI, category-free net reclassification improvement; FIB-4, fibrosis-4 index; MAF-5, metabolic dysfunction-associated fibrosis-5 score; MASLD, metabolic dysfunction-associated steatotic liver disease. *Significance was defined as *P* < 0.05.
