# Supplementary material for: Diagnostic Accuracy of Serum‐Based Fibrosis Scores for Clinically Significant Liver Fibrosis in an East Asian Cohort
Source: Kaohsiung J Med Sci. 2026 Sep 24:e70296. Online ahead of print. doi: 10.1002/kjm2.70296 (PMC13602177; doi:10.1002/kjm2.70296)
Supplement: Supplementary file 2 [file KJM2-9999-e70296-s002.docx]

Supplemental Figure 1. Areas under the receiver operating characteristic curves (AUROCs) are presented for (A) the overall population, (B) male participants, (C) female participants, and the subgroups with (D) metabolic dysfunction-associated steatotic liver disease (MASLD), (E) hepatitis B virus (HBV) infection, and (F) hepatitis C virus (HCV) infection. Predicted probabilities were estimated separately in each of the 50 multiply imputed datasets and averaged across imputations to generate the AUROC estimates.
